# Sonication: A Valuable Technique for Diagnosis and Treatment of Periprosthetic Joint Infections

**DOI:** 10.1155/2013/375140

**Published:** 2013-10-10

**Authors:** D. S. Evangelopoulos, I. P. Stathopoulos, G. P. Morassi, S. Koufos, A. Albarni, P. K. Karampinas, A. Stylianakis, S. Kohl, S. Pneumaticos, J. Vlamis

**Affiliations:** ^1^3rd Department of Orthopaedic Surgery, University of Athens, KAT Hospital, Nikis 2 Street, 14561 Athens, Greece; ^2^Department of Microbiology, KAT Hospital, Nikis 2 Street, 14561 Athens, Greece; ^3^Department of Orthopaedic Surgery, Inselspital, University of Bern, 3010 Bern, Switzerland

## Abstract

*Background*. Periprosthetic joint infection (PJI) is the most severe complication, following joint arthroplasty. Identification of the causal microbial factor is of paramount importance for the successful treatment. *Purpose*. The aim of this study is to compare the sonication fluid cultures derived from joint prosthetic components with the respective periprosthetic tissue cultures. *Methods*. Explanted prosthesis components for suspected infection were placed into a tank containing sterile Ringer's solution and sonicated for 1 minute at 40 kHz. Sonication fluid cultures were examined for 10 days, and the number and identity of any colony morphology was recorded. In addition, periprosthetic tissue specimens (>5) were collected and cultured according to standard practice. The duration of antimicrobial interruption interval before culture sampling was recorded. *Results*. Thirty-four patients composed the study group. Sonication fluid cultures were positive in 24 patients (70.5%). Sixteen of thirty four periprosthetic tissue cultures (47.1%) were considered positive, all revealing the same microbial species with the respective sonication fluid cultures: 3 tissue samples showed polymicrobial infection. All tissue cultures were also found positive by the sonication fluid culture. *Conclusions*. Sonication fluid cultures represent a cheap, easy, accurate, and sensitive diagnostic modality demonstrating increased sensitivity compared to periprosthetic tissue cultures (70.5 versus 47.1%).

## 1. Introduction

Over the last decades the number of total joint replacement procedures has been explosively increased with nearly 800.000 primary THR and TKR performed in the United States and 130.000 in England in 2006 [1, 2]. Their number is expected to rise by 174% and 673%, respectively, by year 2030 [3].

Periprosthetic joint infection (PJI) is the most severe complication, occurring in 0.3 to 1.7% of THR and 0.8 to 1.9% of TKR [4–8], while these rates rise up to 40% after revision surgery [9]. Their associated mortality is estimated to be between 1.0 and 2.7 percent [10–12]. Although they are still considered as an uncommon problem, PJIs pose a heavy social and economic burden with an estimated cost of up to $50.000 per patient and $250 million per year [10, 13], while others estimate that hospital costs per patient requiring revision THR due to infection reach nearly 5 times that of a primary THR [14]. 

Identification of the causal microbial factor is of paramount importance for successful treatment. Staphylococci (*aureus* and coagulase-negative species) account for more than half of the cases of PJI [11, 15], but in up to 20% of the cases more than one microorganism is identified (usually involving methicillin-resistant staphylococci *aureus* or anaerobes) [16]. Existing conventional methods for the detection of the underlying pathogen include microbiological cultures of synovial fluid and intraoperative soft tissue samples. However, 7 to 39% of the cases demonstrate negative cultures, attributed mainly to prior use of antibiotics, formation of protective biofilm at the surface of the implant (which allows proliferation of microorganisms on the prosthesis with no presence at the surrounding soft tissue), and ability of the bacteria to change to a dormant metabolic form with small-colony variants [17–23].

The use of low-intensity ultrasound for the disintegration of biofilm (sonication) on removed implants and the subsequent culture of the sonication fluid is an alternative method for the diagnosis of PJI that has been proved to be more sensitive than conventional periprosthetic tissue cultures [14]. The purpose of the present study was to evaluate the sensitivity of a sonication protocol based on the method of Trampuz et al. [15] in comparison to traditional culture methods for the identification of causal pathogens in PJI following total joint arthroplasties.

## 2. Materials and Methods

Between October 2011 and June 2012, a prospective cohort study was conducted at the authors institution, a University level A Trauma Centre. The study protocol had been approved by the hospital scientific review board. The patients with periprosthetic joint infections composed the study group. Diagnosis of infection was confirmed on the basis of positive laboratory markers, cultures of preoperative aspirates, technetium-methylene diphosphonate (MDP) bone scintigraphy, and intraoperative tissue cultures. Prosthesis or its components (metal fixed or polyethylene mobile components) were removed for diagnosis of infection as a part of a two-stage revision protocol [11]. The first step of the surgical protocol included the explanation of the prosthetic components: an extensive debridement of the infected joint and the implantation of a temporary spacer. The second surgery was performed at a minimum of twelve weeks after first stage operation.

The explanted prosthesis was sent for sonication to detect microorganisms of the biofilm. The patients who had received intravenous antibiotic for at least 24 h in the 10 days before surgery or perioperative antimicrobial prophylaxis before surgery were excluded. Subjects were also excluded if obvious contamination of a removed component occurred in the operating room or fewer than three periprosthetic tissue samples were collected for culture.

Medical records including demographic characteristics; clinical, radiographic, laboratory, histopathological, and microbiological data; type of surgical management; information about the primary arthroplasty and subsequent revisions (if any) and antimicrobial therapy were reviewed and analyzed.

### 2.1. Study Definitions

PJI was considered if one of the following criteria was present: (i) visible purulence of a preoperative aspirate or intraoperative periprosthetic tissue (as determined by the surgeon), (ii) presence of a sinus tract communicating with the prosthesis, (iii) acute inflammation in intraoperative permanent periprosthetic tissue sections by histopathology (as determined by the pathologist), (iv) increased synovial fluid leukocyte count with 1700 leukocytes and/or 65% granulocytes, or (v) microbial growth in intraoperative periprosthetic tissue or sonication fluid of the removed implant. Low-virulence microorganisms, such as coagulase-negative staphylococci or *Propionibacterium acnes*, were considered pathogens if at least one additional (culture-independent) criterion for PJI was fulfilled.

### 2.2. Periprosthetic Cultures

For all patients, at least two intraoperative periprosthetic tissue specimens were retrieved from the bone-cement/bone-prosthesis interface, from sights with obvious inflammatory changes. Tissue specimens were collected in sterile vials and individually homogenized in 3 mL trypticase soy broth for 1 min using mortar and pestle. Tissue homogenate samples were inoculated in 0.1 mL aliquots into aerobic (SBA) and anaerobic sheep blood agar (ASBA) plates and in 1 mL aliquots into thioglycolate broth. The cultures were incubated at 35°C for 10 days. A terminal subculture was performed from all thioglycolate broth specimens on blood agar plates and incubated at 35°C for 5 more days. Each unique colony of isolated microorganisms was identified, and their antimicrobial susceptibility was tested using standard microbiological techniques. Positive tissue cultures were considered those with the same microorganism isolation of at least two periprosthetic tissue samples.

### 2.3. Sonication Fluid Cultures

**Figure 1:**
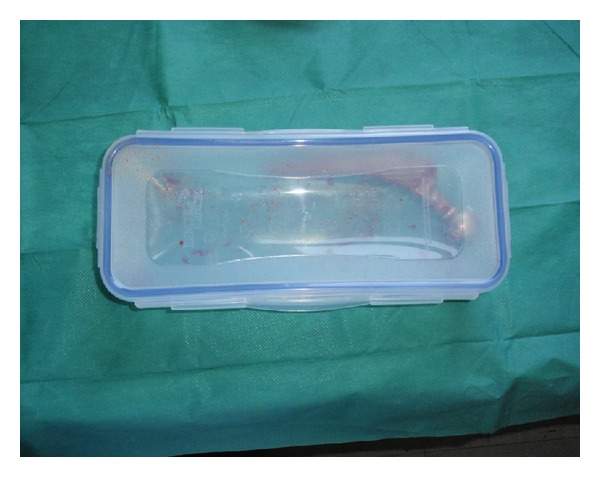


**Figure 2:**
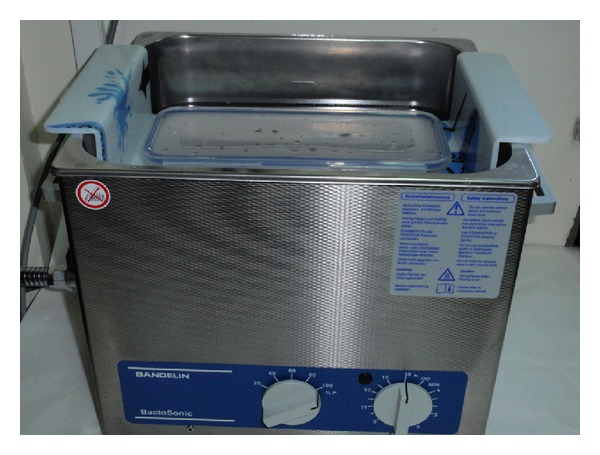


The explanted prosthesis (or its components) was aseptically removed in the operating room and transported to the microbiology laboratory in sterile solid air-tight containers (Lock & Lock; Vertrag AG, Stafa, Switzerland) (Figure 1). Sonication of the implant was performed according to the Trampuz et al. technique [22]. Briefly, sterile Ringer solution (solution volume ranged from 50 to 200 mL depending on the size of implant) was added to the container in a laminar airflow biosafety cabinet to cover 85–90% of the volume of a big sized prosthesis or the entire volume of small sized components. The container with the implant was vortexed for 30 s,  followed by sonication for 1 min (at a frequency of 40 kHz and power density of 0.22 W/cm^2^), as determined by a calibrated hydrophone (type 8103; Bruel and Kjær, Naerum, Denmark). For sonication, ultrasound bath BactoSonic (Bandelin GmbH, Berlin, Germany) was used according to the manufacturer's instructions (http://www.bactosonic.info/) (Figure 2). No differences in frequency or power density were observed at various locations within the ultrasound bath during the study period. The container was subsequently vortexed for an additional 30 s to remove any residual microorganisms and to homogeneously distribute them in the sonication fluid. Aliquots of 0.1 mL sonicate fluid were inoculated into sheep blood agar (SBA) and anaerobic sheep blood agar (ASBA) plates. Additionally, 1 mL of the remaining of sonication fluid was added in 10 mL thioglycollate broth (TGB). The SBA plates and TSB were incubated at 37°C aerobically and the ASBA plates and TGB at 37°C  anaerobically and inspected daily for bacterial growth. Every distinct morphotype colony of microorganisms on plates was enumerated (i.e., number of CFU/mL sonication fluid), identified, and subjected to susceptibility testing by means of routine microbiological techniques.

### 2.4. Negative Controls

Ten consecutive explanted prostheses, revised due to aseptic loosening from patients with no history of previous infection, were included as controls. Following removal, the prosthesis was subjected initially to sonication and then to culture similarly to the prosthesis of the study group.

### 2.5. Statistical Analysis

Comparisons of individual diagnostic tests were performed using the McNemar test. For mixed infections, the test was considered positive if all infecting organisms were detected. Differences were considered significant when *P* values were ≤0.05. All calculations were performed using the statistical software package SPSS (version 13, NC).

## 3. Results

Thirty-four patients undergoing joint prosthesis removal composed the study group. Mean patients' age was 73.1 years (range 54–89 yrs). Erythrocyte sedimentation rate (ESR) and C-reactive protein (CRP) values prior to and six weeks after prosthesis explantation are shown in Table 1.

Sonication fluid cultures were positive in 24 patients (70.5%): 12 coagulase-negative staphylococci (8 methicillin-resistant), 7 *Escherichia coli*, 4 *Staphylococcus aureus* (1 methicillin-resistant), 3 *Proteus spp*., 2 *Pseudomonas spp.*, and 1 *Candida albicans* were identified. In 5 of 24 infected implants (25.2%), mixed infections were found. Different susceptibility testing was received for the same microbial species, especially for CNS (*n* = 4) and *E. coli* (*n* = 2). For periprosthetic tissue cultures, 16 of 34 samples (47.1%) were considered positive, all revealing the same microbial species with the respective sonication fluid cultures: three tissue samples demonstrated polymicrobial infection. All positive periprosthetic tissue cultures were also confirmed by the sonication fluid cultures. In 8 out of 34 patients (23.5%) in whom sonication fluid cultures were negative, the drug interruption interval before culture sampling was less than 7 days.

## 4. Discussion

Diagnosis of PJI is often challenging since many of the typical symptoms of infection can be missing. Several diagnostic modalities such as laboratory tests (white blood cell count, ESR, CRP, Il-6, TNF-*α*, and procalcitonin C), synovial fluid characteristics, histopathological studies of intraoperative samples of periprosthetic tissue, microbiological studies (conventional cultures of five to six intraoperative specimens of periprosthetic tissue), and radiological studies (predominately technetium-methylene diphosphonate (MDP) bone scintigraphy) can be applied to identify the pathogen [23]; however differential diagnosis of low-grade infections can be extremely challenging. Sensitivity and specificity of the aforementioned methods are currently not optimal for single use for the diagnosis of PJI, although several studies have shown that histopathology has a superior sensitivity compared to the microbiological and laboratory exams [24–26]. However, histological diagnosis has the disadvantage that the causative pathogen cannot be identified and so the optimal antibiotic treatment cannot be administered. On the contrary, cultures of periprosthetic tissue and the subsequent antibiogram can provide this essential information. Unfortunately, there is a high rate of negative cultures, a fact that often misleads the clinical decision.

The ability of the bacteria to form biofilms at the surface of implants is a major factor for chronic PJI and one of the main causes for the lack of positive cultures of periprosthetic soft tissue samples obtained intraoperatively [16, 17]. Bacteria can exist in two main forms: the planktonic form characterized by rapid cellular division and the sessile form characterized by slower cellular division, thus being more difficult to grow in cultures [27]. Biofilms are structured consortiums of bacteria in sessile form embedded in a self-produced biopolymer matrix consisting of polysaccharide, protein, and DNA that originate from the microbes. Quite often these consortiums consist of more than one species living in a harmonic way. The matrix provides structural support to the bacteria, facilitating the communication and protecting them from the host's immune system and antibiotics [27, 28].

Utilization of ultrasound to dislodge biofilms from the surface of removed implants (sonication) has been effective in increasing the sensitivity of microbiological studies to identify the underlying pathogen. In a study of 331 patients with THR and TKR comparing sonication to standard tissue culture, the sensitivities of periprosthetic tissue and sonication fluid cultures were 60.8% and 78.5% (*P* < 0.001), respectively, and the specificities were 99.2% and 98.8%, respectively. There were 14 cases of PJI detected by sonication fluid cultures but not by conventional cultures. Of note, in patients receiving antimicrobial therapy within 14 days prior to surgery, the sensitivity of sonication fluid cultures was significantly superior to that of periprosthetic tissue cultures (75.0% versus 45.0%,  *P* < 0.001) [14]. In another similar study with 136 patients undergoing shoulder revision arthroplasty (33 with PJI), sonication fluid cultures were more sensitive than periprosthetic tissue cultures (66.7% versus 54.5%, *P* = 0.046) while specificities were similar to the previous study [29].

 The results of our study show that sonication fluid cultures of microorganisms from removed orthopedic implants are more sensitive than tissue cultures (70.5% and 47.1% resp., *P* < 0.005). The technique is simple and can be performed in most microbiology laboratories. Additionally, as also shown in this study, it demonstrates a higher sensitivity for polymicrobial prosthetic-joint infections compared to intraoperative tissue cultures [15].

Nowadays, even for aseptic loosening, several studies suggest a possible role for bacteria and bacterial biofilm in implant failure [30, 31]. However, since sonication typically yields high numbers of organisms, one has to be aware of false positive results due to explanted prostheses contamination at the operating room or the microbiology laboratory. Further studies are required to quantify the number of microorganisms in sonicate fluid and assess the boundaries between PJI and contamination of explanted prosthesis following aseptic loosening [15].

## 5. Conclusions

 Sonication of removed arthroplasty components using low-frequency ultrasound (35–40 kHz) was shown to improve microbiologic diagnosis of periprosthetic infections. Sonication fluid culture represents a cheap, easy, accurate, and sensitive diagnostic modality compared to periprosthetic tissue cultures. Staphylococci (especially coagulase-negative staphylococci) were the predominant pathogen, followed by *E. coli*.

## Figures and Tables

**Table 1:** Sensitivity and ESR-CRP values prior to and six weeks after prosthesis explantation.

	Tissue culture	Sonication fluid culture		ESR (mean ± SD)	CRP (mg/L) (mean ± SD)
No. of pts	16/34	24/34	prior to explantation	56.4 ± 36.2	160.8 ± 45.7
Sensitivity	47.1%	70.5%	6 w after explantation	29.1 ± 6.3	8.8 ± 4.8

## References

[1] del Pozo JL, Patel R (2009). Infection associated with prosthetic joints. *The New England Journal of Medicine*.

[2] Cataldo MA, Petrosillo N, Cipriani M, Cauda R, Tacconelli E (2010). Prosthetic joint infection: recent developments in diagnosis and management. *Journal of Infection*.

[3] Kurtz S, Ong K, Lau E, Mowat F, Halpern M (2007). Projections of primary and revision hip and knee arthroplasty in the United States from 2005 to 2030. *Journal of Bone and Joint Surgery A*.

[4] Pulido L, Ghanem E, Joshi A, Purtill JJ, Parvizi J (2008). Periprosthetic joint infection: the incidence, timing, and predisposing factors. *Clinical Orthopaedics and Related Research*.

[5] Choong PFM, Dowsey MM, Carr D, Daffy J, Stanley P (2007). Risk factors associated with acute hip prosthetic joint infections and outcome of treatment with a rifampinbased regimen. *Acta Orthopaedica*.

[6] Phillips JE, Crane TP, Noy M, Elliott TSJ, Grimer RJ (2006). The incidence of deep prosthetic infections in a specialist orthopaedic hospital. A 15-year prospective survey. *Journal of Bone and Joint Surgery B*.

[7] Jämsen E, Huhtala H, Puolakka T, Moilanen T (2009). Risk factors for infection after knee arthroplasty a register-based analysis of 43,149 cases. *Journal of Bone and Joint Surgery A*.

[8] Peersman G, Laskin R, Davis J, Peterson M (2001). The insall award paper: infection in total knee replacement: a retrospective review of 6489 total knee replacements. *Clinical Orthopaedics and Related Research*.

[9] Widmer AF (2001). New developments in diagnosis and treatment of infection in orthopedic implants. *Clinical Infectious Diseases*.

[10] Zimmerli W (2006). Prosthetic-joint-associated infections. *Best Practice and Research: Clinical Rheumatology*.

[11] Zimmerli W, Trampuz A, Ochsner PE (2004). Prosthetic-joint infections. *The New England Journal of Medicine*.

[12] Ahnfelt L, Herberts P, Malchau H, Andersson GBJ (1990). Prognosis of total hip replacement. A Swedisch multicenter study of 4,664 revisions. *Acta Orthopaedica Scandinavica, Supplementum*.

[13] Sculco TP (1995). The economic impact of infected joint arthroplasty. *Orthopedics*.

[14] Bozic KJ, Ries MD (2005). The impact of infection after total hip arthroplasty on hospital and surgeon resource utilization. *Journal of Bone and Joint Surgery A*.

[15] Trampuz A, Piper KE, Jacobson MJ (2007). Sonication of removed hip and knee prostheses for diagnosis of infection. *The New England Journal of Medicine*.

[16] Marculescu CE, Cantey JR (2008). Polymicrobial prosthetic joint infections: risk factors and outcome. *Clinical Orthopaedics and Related Research*.

[17] Berbari EF, Marculescu C, Sia I (2007). Culture-negative prosthetic joint infection. *Clinical Infectious Diseases*.

[18] del Pozo JL, Patel R (2007). The challenge of treating biofilm-associated bacterial infections. *Clinical Pharmacology and Therapeutics*.

[19] Malekzadeh D, Osmon DR, Lahr BD, Hanssen AD, Berbari EF (2010). Prior use of antimicrobial therapy is a risk factor for culture-negative prosthetic joint infection. *Clinical Orthopaedics and Related Research*.

[20] Holinka J, Bauer L, Hirschl AM, Graninger W, Windhager R, Presterl E (2011). Sonication cultures of explanted components as an add-on test to routinely conducted microbiological diagnostics improve pathogen detection. *Journal of Orthopaedic Research*.

[21] Neut D, van der Mei HC, Bulstra SK, Busscher HJ (2007). The role of small-colony variants in failure to diagnose and treat biofilm infections in orthopedics. *Acta Orthopaedica*.

[22] Donlan RM (2005). New approaches for the characterization of prosthetic joint biofilms. *Clinical Orthopaedics and Related Research*.

[23] Osmon DR, Berbari EF, Berendt AR (2013). Diagnosis and management of prosthetic joint infection: clinical practice guidelines by the infectious diseases society of America. *Clinical Infectious Diseases*.

[24] Fink B, Makowiak C, Fuerst M, Berger I, Schäfer P, Frommelt L (2008). The value of synovial biopsy, joint aspiration and C-reactive protein in the diagnosis of late peri-prosthetic infection of total knee replacements. *Journal of Bone and Joint Surgery B*.

[25] Müller M, Morawietz L, Hasart O, Strube P, Perka C, Tohtz S (2008). Diagnosis of periprosthetic infection following total hip arthroplasty—evaluation of the diagnostic values of pre- and intraoperative parameters and the associated strategy to preoperatively select patients with a high probability of joint infection. *Journal of Orthopaedic Surgery and Research*.

[26] Tohtz SW, Müller M, Morawietz L, Winkler T, Perka C (2010). Validity of frozen sections for analysis of periprosthetic loosening membranes. *Clinical Orthopaedics and Related Research*.

[27] Costerton JW, Stewart PS, Greenberg EP (1999). Bacterial biofilms: a common cause of persistent infections. *Science*.

[28] Høiby N, Bjarnsholt T, Givskov M, Molin S, Ciofu O (2010). Antibiotic resistance of bacterial biofilms. *International Journal of Antimicrobial Agents*.

[29] Piper KE, Jacobson MJ, Cofield RH (2009). Microbiologic diagnosis of prosthetic shoulder infection by use of implant sonication. *Journal of Clinical Microbiology*.

[30] Hoenders CSM, Harmsen MC, van Luyn MJA (2008). The local inflammatory environment and microorganisms in “aseptic” loosening of hip prostheses. *Journal of Biomedical Materials Research B*.

[31] Tamaki Y, Takakubo Y, Goto K (2009). Increased expression of toll-like receptors in aseptic loose periprosthetic tissues and septic synovial membranes around total hip implants. *Journal of Rheumatology*.

